# Data collection in pandemic times: the case of the Belgian COVID-19 health surveys

**DOI:** 10.1186/s13690-023-01135-x

**Published:** 2023-07-04

**Authors:** Elise Braekman, Rana Charafeddine, Finaba Berete, Helena Bruggeman, Sabine Drieskens, Lydia Gisle, Lize Hermans, Johan Van der Heyden, Stefaan Demarest

**Affiliations:** Department Epidemiology and public health, Sciensano, Brussels, Belgium

**Keywords:** COVID-19, Coronavirus, General population, Web surveys, Non-probability surveys, Longitudinal data collection, Participation, Belgium

## Abstract

**Background:**

Survey data were needed to assess the mental and social health, health related behaviors and compliance with preventive measures of the population during the COVID-19 pandemic. Yet, the pandemic challenged classical survey methods. Time and budgetary constraints at the beginning of the pandemic led to ad hoc recruitment of participants and easily manageable data collection modes. This paper describes the methodological choices and results in terms of participation for the COVID-19 health surveys conducted in Belgium.

**Methods:**

The COVID-19 health surveys refer to a series of ten non-probability web surveys organized between April 2020 and March 2022. The applied recruitment strategies were diverse including, amongst others, a launch through the website and the social media of the organizing research institute. In addition, the survey links were shared in articles published in the national press and participants were requested to share the surveys in their network. Furthermore, participants were asked consent to be re-contacted for next survey editions using e-mail invitations.

**Results:**

These mixed approaches allowed to reach a substantial number of participants per edition ranging from 49339 in survey 1 to 13882 in survey 10. In addition, a longitudinal component was created; a large share of the same individuals were followed up over time; 12599 participants completed at least 5 surveys. There were, however, sex, age, educational level and regional differences in participation. Post-stratification weighting on socio-demographic factors was applied to at least partly take this into account.

**Conclusion:**

The COVID-19 health surveys allowed rapid data collection after the onset of the pandemic. Data from these non-probability web surveys had their limitations in terms of representativeness due to self-selection but were an important information source as there were few alternatives. Moreover, by following-up the same individuals over time it was possible to study the effect of the different crisis phases on, amongst others, the mental health. It is important to draw lessons from these experiences: initiatives in order to create a survey infrastructure better equipped for future crises are needed.

**Supplementary Information:**

The online version contains supplementary material available at 10.1186/s13690-023-01135-x.

**Table:** 

Text box 1. Contributions to literature
• Surveys aiming to rapidly evaluate the impact of the COVID-19 pandemic on the population faced budget and time challenges and dependent on existing survey infrastructure. Non-probability web surveys prone to self-selection were commonly used.
• In Belgium, the COVID-19 health surveys, 10 cross-sectional non-probability web surveys with a longitudinal component, were organized. Using diverse recruitment strategies a substantial number of participants were reached. Yet, significant socio-demographic differences in the participant pool were found.
• Non-probability web surveys were an important information source when probability surveys were impossible. Initiatives to improve the survey infrastructure to be better prepared for future crises are important.

## Background

The COVID-19 pandemic caused by the severe acute respiratory syndrome coronavirus (SARS-CoV-2) has had a tremendous impact on people’s lives due to the uncertainties and fears that were associated with the outbreak of the virus [1–4]. In addition, people’s lives were affected by nation-wide preventive measures adopted to reduce the transmission of the virus including physical distancing and lockdowns during the most critical phases of the crisis. In order for decision makers to manage the outcomes of the crisis, close epidemiological monitoring was of utmost importance. Besides the need of surveillance data on the number of COVID-19 infections, hospitalizations, deaths and vaccination, data on how the population experienced this long-lasting crisis were also crucial [2, 5, 6]. Information on the impact of the crisis on mental health in terms of anxiety, depression, psychological distress, loneliness, etc. [1–4, 7] and on health related behaviors e.g. physical activity, sedentary behavior and nutritional habits [8–11] was crucial. Moreover, data on the knowledge, perceptions and adherence to the preventive measures were highly valuable as the effectivity of these measures was largely depended on the compliance of the population [5, 12–16].

These data could be collected using population-based surveys but the pandemic imposed some specific challenges. In the first weeks of the pandemic surveys needed to be developed and organized rapidly to assess the impact of the most severe lockdowns [13, 14]. Related to this, the first surveys had to be organized with limited budgets since there was no time to request large funding [13]. In addition, due to physical distancing surveys could not be administered face-to-face [13]. Lastly, it was expected that this public health crisis and the associated psychosocial effects would impact the population in the longer run. Consequently, it was advisable to monitor the population over time using multiple surveys [4]. These aspects impacted the methodological choices of population-based surveys set up during the COVID-19 pandemic. More specifically, they guided choices related to the mode of data collection, the sampling approach and the type of observational study design.

The face-to-face mode is traditionally seen as the ‘gold standard’ for data collection. An interviewer on the doorstep is effective to get high participation rates, even in more difficult to reach population groups [17, 18]. Moreover interviewers can clarify questions, probe for responses and keep participants motivated through long questionnaires [19, 20]. Nevertheless, confinement and quarantine periods made this impossible. An alternative interviewer-administered mode often considered in COVID-19 times was the telephone mode [21]. It is, however, expensive and time consuming as interviewers need to be hired and a specific infrastructure needs to be developed. The web mode has some particular advantages over these interviewer-administered modes and over the paper-and-pencil self-administered mode such as 1) it is completely self-administered which not only reduces the chance for social desirability bias but also limits the chance to spread the virus; 2) it is, contrary to a paper-and-pencil mode, computer-assisted and therefore presents the advantage of automatic data entry and automatic branching logic; 3) costs are lower compared to the paper-and-pencil, face-to-face and telephone mode and 4) web surveys can be developed and implemented rapidly with easy-to-use software. Researchers therefore turned to online data collection in pandemic times [6, 21–24].

Although, there are many advantages to web data collection, there are certain risks too. Web surveys exclude people without internet access or skills to use the internet by default to complete the survey [25]. Recent data showed that 6% of the Belgian population (16-74 years old) never used the internet and about 40% has low or no digital skills [26]. A recent Belgian study showed that web surveys are prone to low response rates, especially among elderly, lower educated people, people with a migration background and people living alone [18]. Moreover, internet user and non-users might have a different health profile and weighting for demographic variables does not eliminate the observed health differences [27].

Another important methodological choice, often linked to the mode of data collection, is between probability and non-probability sample surveys. In the first approach each member of the population has a known and positive chance to be selected which enables statistical inference, non-probability sample surveys refer to all other types of surveys [28]. Probability sampling is preferred over non-probability sampling, especially for estimating population characteristics [25, 29, 30]. However, setting up surveys in new probability samples can be an expensive and time consuming process [21]. Many COVID-19 web surveys were therefore organized in non-probability samples [23, 24]. These non-probability web surveys were either quota samples from commercial panels [12, 15] or convenience samples with self-selected participants [13, 31–35]. By using e-mail, website or social media announcements for convenience surveys thousands of participants can be reached instantly [28]. Nevertheless, the validity of research findings depends more on the representativeness capacity than on the participant number [21].

Lastly, not all types of observational study designs are suited to monitor the impact of the evolving situation over time. Integrating COVID-19 surveys within existing longitudinal surveys having pre-pandemic information was recommend for this [36–38]. In these types of surveys, the same participants were questioned on the same topic before and during the pandemic using (ideally) the same data collection mode. This design allowed to study causal relationships at both the individual and group level. Yet if no existing longitudinal surveys could be used, new longitudinal surveys to follow-up the same individuals were considered valuable as well [4, 39, 40]. The drawback, however, is that comparisons with pre-COVID-19 survey data were hampered due to other methodology approaches in the new surveys.

This manuscript discusses how researchers at Sciensano, the Belgian institute of health, dealt with these challenges in organizing the COVID-19 health surveys. This is a series of ten repeated online surveys that ran between April 2020 and March 2022. These surveys had as objective to monitor the general adult population on health related topics that were relevant for policy makers and supported them in fighting the pandemic and its effects, in the medium and long term. The main objectives of this manuscript are:

- to describe the methodology used in the COVID-19 health surveys;
- to provide the outcomes of the COVID-19 health surveys in terms of participation and sample composition;
- to discuss the benefits and pitfalls of the applied methodology and provide directions for future research.

## Methods

Over a period of two years and therefore in different phases of the pandemic, ten online COVID-19 health surveys were organized in Belgium. The general methodology is discussed below. The elements that differed from survey to survey are presented in Table 1. Systematic methodological information about the surveys based on The Checklist for Reporting Results of Internet E-Surveys (CHERRIES) [41] can be found in Additional file 1. All ten surveys were approved by the ethical committee of the University Hospital of Ghent. Before participants could participate to the survey, they had to indicate that they lived in Belgium and were at least 18 years old. Furthermore, in all surveys participants had to provide consent to six terms and conditions including voluntary participation, confidentiality of the data and the right to withdraw at any time in accordance with the General Data Protection Regulation (GDPR) and the Declaration of Helsinki.

**Table 1:** COVID-19 health surveys by timing and crisis phase, recruitment, themes and participant number, Belgium 2020-2022

**Timing & phase in the crisis**	**Recruitment strategy**	**Main themes included**	**Number of participants**
**Survey 1**			
April 2 – 9, 2020 High level of new hospital admissions per day^a^ Severe restrictions	River sampling trough Sciensano and press Snowball sampling via participants and Sciensano employees	Mental, social and general health Health related behaviors (consumption of alcohol, tobacco and illegal drugs) COVID-19 symptoms, tests and infections Contact with health care Perceived knowledge and compliance to measures Telework Information sources used for COVID-19 Trust in authorities	49334
**Survey 2**			
April 16 – 24, 2020 High level of new hospital admissions per day Severe restrictions	River sampling trough Sciensano, press, local community organizations, health insurance funds and elderly organizations Recruitment of previous participants via e-mail Snowball sampling via participants and Sciensano employees Offline recruitment via Coronavirus press conference	Mental and social health Quality of life Health related behaviors (physical activity, nutritional habits, nutritional status, consumption of alcohol, tobacco, illegal drugs and sedatives) COVID-19 tests and infections Domestic violence Perceived knowledge and compliance to measures Information sources used for COVID-19 Trust in authorities Telework and measures at work taken to reduce infections Financial situation	42895
**Survey 3**			
May 28 – June 5, 2020 Low level of new hospital admissions per day Moderate restrictions	River sampling trough Sciensano, press, local community organizations, health insurance funds and elderly organizations Recruitment of previous participants via e-mail Snowball sampling via Participants and Sciensano employees	Mental and social health Chronic conditions COVID-19 tests and infections Perceived knowledge and compliance to measures Telework and measures at work Financial situation and food security Health literacy	33913
**Survey 4**			
September 24 – October 2, 2020 Moderate level of new hospital admissions per day Light restrictions	River sampling trough Sciensano, press, local community organizations, health insurance funds and elderly organizations Recruitment of previous participants via e-mail Snowball sampling via participants and Sciensano employees Recruitment of diabetes patients via patient organization	Mental and social health Health related behaviors (consumption of alcohol, tobacco, illegal drugs and sedatives) Chronic conditions Impact COVID-19 on diabetes patients COVID-19 tests and infections Perceived knowledge and compliance to measures Telework and measures at work Attitude towards contact tracing centers and contact tracing app Attitude towards COVID-19 vaccination Trust in institutions COVID-19 related health literacy Impact on life domains Interpersonal trust	30845
**Survey 5**			
December 3 –11, 2020 High level of new hospital admissions per day Moderate restrictions	River sampling trough Sciensano, press, local community organizations, health insurance funds and elderly organizations Recruitment of previous participants via e-mail Snowball sampling via participants and Sciensano employees Offline recruitment via Coronavirus press conference	Mental and social health Health related behaviors (gambling habits, consumption of alcohol, tobacco, illegal drugs and sedatives) COVID-19 tests and infections Contact with health care Perceived knowledge and compliance to measures Telework Attitude towards (COVID-19) vaccination Trust in institutions Food security and difficulties paying health care expenses	29855
**Survey 6**			
March 18 – 25, 2021 High level of new hospital admissions per day Moderate restrictions	River sampling trough Sciensano, press, local community organizations, health insurance funds and elderly organizations Recruitment of previous participants via e-mail Snowball sampling via participants and Sciensano employees Offline recruitment via Coronavirus press conference	Mental and social health Health related behaviors (physical activity, nutritional habits, nutritional status and consumption of alcohol) Chronic conditions Frailty among elderly Quality of life COVID-19 tests and infections Compliance to measures COVID-19 vaccination adherence Impact on life domains Domestic violence	20410
**Survey 7**			
June 10 – 20, 2021 Low level of new hospital admissions per day Light restrictions	River sampling trough Sciensano, press, local community organizations, health insurance funds, elderly organizations, sports federations, higher education institutes and young adult organizations Recruitment of previous participants via e-mail Snowball sampling via participants and Sciensano employees	Mental and social health Chronic conditions Quality of life COVID-19 vaccination adherence Side effects related to COVID-19 vaccination Impact on life domains	17774
**Survey 8**			
October 4 – 18, 2021 Moderate level of new hospital admissions per day Light restrictions	River sampling trough Sciensano, press, local community organization, health insurance funds, elderly organizations, sports federations, higher education institutes and young adult organizations Recruitment of previous participants via e-mail Snowball sampling via participants and Sciensano employees	Mental and social health Health related behaviors (consumption of alcohol, tobacco and illegal drugs) Chronic conditions Frailty among elderly Quality of life COVID-19 infections and symptoms Compliance to measures COVID-19 vaccination adherence Perceptions of how well institutions handled the crisis Impact on life domains	17347
**Survey 9**			
December 13 –23, 2021 High level of new hospital admissions per day Moderate restrictions	River sampling trough Sciensano, press, local community organizations, health insurance funds, elderly organizations, sports federations, higher education institutes and young adult organizations Recruitment of previous participants via e-mail Snowball sampling via Participants and Sciensano employees	Mental and social health COVID-19 (self-)tests, infections and symptoms Compliance to measures Attitude on the effectiveness of measures COVID-19 vaccination adherence Attitude towards COVID-19 vaccination Perceived knowledge on vaccination Impact on life domains Use of digital health care platforms Digital health literacy	22354
**Survey 10**			
March 17 – 27, 2022 High level of new hospital admissions per day Light restrictions	River sampling trough Sciensano, press, local community organizations, health insurance funds, elderly organizations, sports federations, higher education institutes and young adult clubs Recruitment of previous participants via e-mail Snowball sampling via participants and Sciensano employees	Mental and social health Health related behaviors (consumption of alcohol, tobacco, illegal drugs and sedatives) COVID-19 (self-)tests and infections Presence of long COVID-19 Telework Impact on life domains Worries about COVID-19 pandemic, climate change, war in Ukraine… Food security and difficulties paying health care expenses	13882

^a^The following categorization was applied: low <65 new hospital admissions a day, moderate =65-149 new hospital admissions a day, and high ≥150 new hospital admissions a day [42]

### Timing 

The first COVID-19 health survey was launched three weeks after the first restrictive measures were put in place. The subsequent surveys followed at regular time intervals. Table 1 provides more details about the timing and phases of the crisis in which the different surveys were organized. The phase of the crisis refers to the epidemiological situation and the severity of the restrictions put in place at that time. For the epidemiological situation, the number of new hospital admissions due to COVID-19 as presented on the Dashboard of Sciensano were taken into account [42]. In line with the Belgian “Coronabarometer”, we considered <65 new hospital admissions a day as low, between 65-149 new hospital admissions a day as moderate and ≥150 new hospital admissions a day as high” [43]. For the severity of the restrictions, the measures as presented on the official website of the government served as the reference [44]. Three distinctions were made:

- Severe restrictions included i.a. closure of non-essential shops, bars, restaurants and schools, telework was the norm, non-essential movements and social contacts outside the household were strictly limited.
- Moderate restrictions included i.a. non-essential shops, bars, restaurants and schools were closed or open with restrictions, telework was the norm but combined with office days, non-essential movements and social contacts outside the household, though often with restrictions, were allowed.
- Light restrictions referred to periods where i.a. non-essential shops, bars, restaurants and schools were fully open, telework was at most a recommendation and there were no or only limited rules for non-essential movements and social contacts outside the household.

### Recruitment strategy

A non-probability sampling approach was used for the COVID-19 health surveys. In crisis times, a permission from the Belgian national register to draw a new probability sample could be received in short time. However, this registry does not contain any e-mail addresses and consequently sampled individuals can only be invited via post which is time and cost inefficient. Relying on a probability-based sample established prior to the pandemic was also not possible because there was no permissions to re-contact participants from a previous large-scale probability survey such as the Belgian health interview survey 2018. Moreover, it was impossible to use members of a probability-based panel as these panels did not exist in Belgium when the crisis started.

The recruitment strategies can be summarized as follows:

- River sampling: this refers to recruiting participants by putting an invitation to complete a survey on a website, a social media page, etc. where it is likely to be noticed by members of the target population [30]. Lehdonvirta et al. describe it poetically as “researchers dipping into the traffic flow of a website, catching some of the users floating by“. All COVID-19 health surveys were announced on the website, the Twitter® and LinkedIn® of Sciensano. In addition, they were announced via (online) articles of national press organizations because each survey had a press release. Starting from the second survey, local community organizations, health insurance funds and senior citizens organizations were asked to share survey invitations through their website, and social media. Starting from the seventh survey also sports federations, higher education institutes and young adult organizations received an invitation to spread the survey to attract more youngsters.
- Snowball sampling: this refers to participants recruiting new participants from their network [45]. The name derives from the idea that the sample appears to grow like a rolling snowball. In the case of the COVID-19 health surveys, participants were asked to share the survey invitation as widely as possible among their friends, family and colleagues via e-mail and social media. In addition, Sciensano employees were asked to share the surveys among their personal contacts.
- Recruitment of previous participants: starting from the second survey, invitation e-mails were sent to a list of previous participants who agreed in a given survey that their e-mail address could be kept for this purpose. The e-mail invitations were developed and sent using software of Tripolis®. At the time of the last COVID-19 health survey, 50423 former participants received an invitation. By inviting previous participants, a follow-up was also possible. The data of people who participated in multiple waves of the COVID-19 health surveys was linked between waves. Their e-mail address combined in some cases with some background information and the four last digits of their phone number served for this linkage as there was no other unique identifier. Participants gave consent for this approach. For privacy reasons, the e-mail addresses were separated immediately from the datasets used for the analyses.
- Offline recruitment: in addition to online recruitments, there was also offline recruitment for some COVID-19 health surveys. The surveys were announced during the Coronavirus press conferences organized on a regular basis by the National Crisis Center to inform the population about the epidemiological situation. Some surveys were also mentioned in offline media news.

The specific approaches used per survey can be found in Table 1. The recruitment strategies were continuously adapted in order to try to keep participation high and to attract a sample diverse in terms of socio-demographic characteristics. Materials, such as visuals, social media messages and e-mails, were developed together with internal communication experts.

In order to partly correct for bias associated with this sampling and recruitment strategy, post-stratification weights were applied in the analysis of the data. For what concerns sex, age group and province, information on the composition of the population on January 1^st^, 2019 as calculated by Statbel, the Belgian Statistical Office, was used. To address unequal participation by educational level, weights were adapted according to the information on educational level collected in the context of the Labor Force Survey 2018 [46]. Two educational levels were distinguished: “higher secondary education or lower” and “higher education”. For topics related to COVID-19 vaccination, specific weights were used taking into account the vaccination status of the population at the moment of the survey.

### Web questionnaire

#### Design

All surveys were developed using LimeSurvey® version 3. This is an open source tool that makes it possible to create large-scale sophisticated surveys in a short period of time. The mean completion time of the COVID-19 health surveys ranged between 11 minutes and 20 seconds (eighth survey) and 17 minutes and 27 seconds (fifth survey). The questionnaires could be completed in the three national languages of Belgium (Dutch, French and German) and in English.

#### Content

The questionnaires were developed in consultation with public health experts and policy makers. As much as possible validated instruments were used, such as the ones included in the national health interview survey of 2018. An overview of the health themes covered in the different COVID-19 health surveys is provided in Table 1. Overall, there were five broad domains included in the COVID-19 health surveys, with the two first domains being considered as the core.

- The indirect effects of the COVID-19 crisis on various aspects of health (mental health, social health, health related behaviors and health care consumption).
- Preventive measures taken to reduce the number of transmissions.
- The direct impact of the COVID-19 virus on health (contraction of COVID-19 and its consequences).
- The indirect effects of the COVID-19 crisis on other life domains (e.g. financial and work situation)
- Various aspects that may have influenced the above mentioned outcomes (e.g. education level, employment situation, income, presence of chronic diseases and personality characteristics).

## Results

### Participation & sample composition

**Fig. 1:**
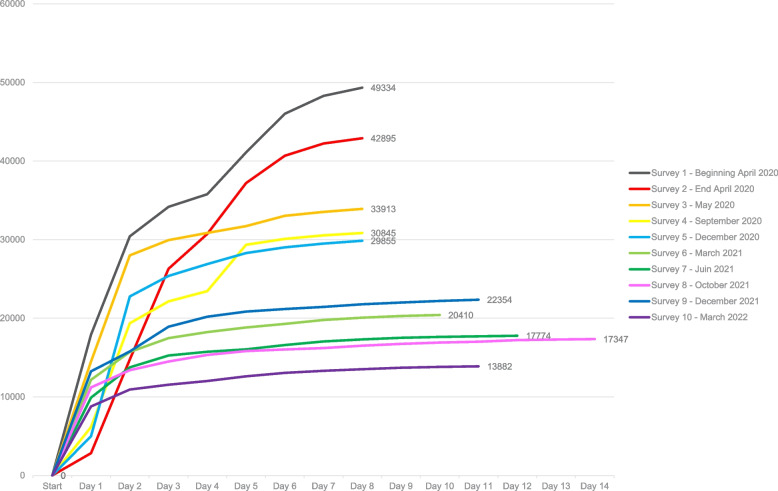
Number of participants by completion day per survey, COVID-19 health surveys, Belgium 2020-2022

Figure 1 displays the total number of participants per survey and gives an overview of the cumulative number of people that completed the survey per day. A participant was defined as a person who agreed with the informed consent and completed minimally the questions on birth year, sex and postal code. Two general trends can be identified. Firstly, the participation decreased consistently over time. The highest participation was reached in the early days of the crisis in Belgium (surveys 1 and 2). The only exception to the decreasing trend was the ninth survey which was organized between 13 and 23 December 2021 and had more participants than the sixth till the eighth survey. A second general trend is that the majority of participants completed the survey within two days after the launch of the survey (>60%). The only exception is the second survey where a large share of the participants completed the survey on the third day too.

Table 2 shows the unweighted sample distribution of the surveys versus the population distribution in terms of sex, age group, education level and region. It also presents the distribution among participants who completed at least 5 surveys and provided consent to link their data collected in the different surveys. This group of people can be followed-up longitudinally and is called the “cohort”.

**Table 2:** Unweighted sample distribution of the surveys versus population distribution, COVID-19 health surveys, Belgium 2020-2022

	Survey 1 - Beginning of April 2020 (*n*=49334)	Survey 2 - End of April 2020 (*n*=42895)	Survey 3 - May 2020 (*n*=33913)	Survey 4 - September 2020 (*n*=30845)	Survey 5 - December 2020 (*n*=29855)	Survey 6 - March 2021 (*n*=20410)	Survey 7 - June 2021 (*n*=17774)	Survey 8 - October 2021 (*n*=17347)	Survey 9 - December 2021 (*n*=22354)	Survey 10 - March 2022 (*n*=13882)	Cohort (*n* = 12599)^a^	Population distribution 2020^b,c^
	**%**	**%**	**%**	**%**	**%**	**%**	**%**	**%**	**%**	**%**	**%**	%
**Sex**												
Men	31.8	31.6	34.9	36.5	38.7	35.6	35.8	36.2	36.5	36.0	35.8	48.8
Women	68.2	68.4	65.1	63.5	61.3	64.4	64.2	62.8	63.5	64.0	64.1	51.2
**Age group**												
18-24	5.3	3.3	3.3	2.3	2.5	1.7	3.3	5.8	4.6	2.8	1.0	10.1
25-34	19.7	15.7	14.2	12.4	11.9	9.8	10.6	9.3	10.3	7.9	8.5	16.2
35-44	24.4	22.6	20.3	20.4	19.3	17.9	16.6	15.5	18.4	14.1	16.4	16.2
45-54	21.2	22.9	21.1	21.9	20.9	20.8	20.0	19.5	20.6	19.6	20.5	17.0
55-64	18.1	21.4	23.0	23.7	24.6	24.4	24.7	24.3	23.3	25.4	26.1	16.6
65-74	9.5	11.9	15.6	16.5	17.9	21.2	20.6	21.0	19.2	24.2	23.0	12.7
75+	1.8	2.2	2.6	2.9	3.0	4.1	4.3	4.6	3.6	6.2	4.5	11.3
**Highest diploma**												
Secondary diploma or less	27.6	26.8	25.2	25.3	24.4	24.8	25.5	27.7	27.5	26.1	25.3	58.9
Higher education	69.4	69.8	71.5	71.9	72.9	72.6	72.1	70.0	69.8	71.5	74.7	41.1
**Region**												
Flemish Region	64.5	50.8	67.8	68.5	69.3	65.4	64.5	64.6	65.0	65.7	63.1	58.2
Brussels Capital Region	9.9	11.3	8.4	9.3	8.9	9.9	9.1	9.3	8.7	9.3	9.9	10.3
Walloon Region	25.7	37.9	23.8	22.2	21.8	24.7	26.4	26.0	26.3	25.0	26.7	31.6

^a^Cohort equals all participants who completed at least 5 surveys and provided consent to link their data collected in the different surveys

^b^The sex, age and regional distribution is based on the distribution of the Belgian registered population aged 18 and older on 01/01/2020, source Statistics Belgium [47]

^c^The education distribution is based on the distribution of the active and inactive population aged 20 till 64 according to the Labor Force Survey [48]

The distribution by sex was the least favorable in the first two surveys (with about 32% males versus 68% females). The sex balance was slightly better in the next surveys and among the cohort participants. The distribution, however, remained highly different from the distribution in the Belgian population (49% males versus 51% females). When assessing the age distributions, some general trends can be observed. In the first survey, there was, compared to the general population distribution, an underrepresentation of the youngest (18-24 years) and the two oldest age groups (65-74 years, 75+ years) and an overrepresentation of the age groups between 25 and 64 years, notably of the age group 35-44 years. Throughout the next editions of the survey, there was a decline in the number of participants from the younger age groups between 18 and 44 years. An exception to this general trend occurred in the seventh and eighth survey where there was an increase in the young participants between 18 and 24 years. The proportional number of participants between 55 and 74 years old increased throughout the surveys. In all COVID-19 health surveys, there was an underrepresentation of the youngest (18-24 years) and oldest age groups (75+). When comparing the distribution of the cohort participants with the distribution in the general population, we observe an underrepresentation of the youngest (18-34 years) and oldest age groups (75+ years) and an overrepresentation of the 45-74 year olds.

The education distribution remained fairly constant during all COVID-19 health surveys and was strongly biased (with about 70% high educated people). Among the cohort participants it was even slightly more skewed with almost 75% high educated people. For comparison, in the general population aged 20 till 64 years only 41% has a degree of higher education. Lastly, the distribution by region remained roughly constant throughout the surveys (on average 66% participants from the Flemish Region, 9% participants from the Brussels Capital Region and 25% participants from the Walloon Region), with the only exception of the second survey where relatively more participants of the Walloon Region (38%) and less participants from the Flemish Region (51%) were counted. The cohort distribution is similar to the survey distributions. When comparing these distributions to the actual distribution in the Belgian population, an overrepresentation of people from the Flemish Region (58% of the population) and an underrepresentation of people from the Walloon Region (32% of the population) can be observed.

## Discussion

The beginning days of the COVID-19 pandemic impacted all aspects of life, including the way surveys were organized [49]. Ongoing and planned studies using face-to-face data collection needed to adjust their fieldwork [50–52]. New surveys aiming to rapidly evaluate the impact of the pandemic faced challenges and were dependent on the existing survey infrastructure of their country. This manuscript described the methodology of the COVID-19 health surveys, a series of 10 non-probability web surveys in Belgium aiming to monitor the general population after the onset of the pandemic. Recommendation to recruit a demographically balanced participant pool were taken into account for these surveys. Informal partnerships were set up with trustworthy organizations such as local community organizations, health insurance funds, young adults and elderly organizations, etc. to build trust among different population groups [6]. Moreover, the recruitment channels (e.g. e-mail, social media, press, etc.) and networks were divers [6, 22, 28]. In addition, extra efforts were made for next survey editions when realizing that some population groups were not enough represented (e.g. substantial efforts were made starting from the seventh survey to attract more young adults).

### Principal findings in terms of participation

In the beginning of the pandemic the number of participants was the highest; the first survey organized within three weeks after the first restrictions were put in place had 49334 participants and the second survey organized two weeks later had 42895 participants. Even though the number of participants decreased throughout time it remained high: the last survey ended with 13882 participants. The participation trend does not follow the severity of the epidemiological situation as some surveys were organized in other critical phases, but had nevertheless much less participants than the first surveys. The declining participation rate may have several reasons. In general, at the beginning of the pandemic, the news and people’s own thoughts and lives were dominated by COVID-19 making the survey topic highly salient. Moreover, the first surveys were organized in strict lockdown periods which gave people time to complete the survey. The two former reasons resulted in a wide dissemination of the COVID-19 health surveys by the press in the beginning of the pandemic while the media attention decreased for later surveys. The only exception to the decreasing trend was the ninth survey which was organized between 13 and 23 December 2021. Possible explanations might be that people had more time during the Christmas period, that the communication materials were more clear after updating them and that the survey was hold in a period with a high number of infections. A declining participation trend over time is also seen in other repetitive COVID-19 surveys [13, 40].

The majority of participants of all COVID-19 health surveys were reached the first and second day after the launch of the surveys. This indicates that the surveys were mainly shared within the first days after the launch and that people completed the survey almost immediately after viewing the link to the survey on a website, a social media page or an invitation e-mail. The only exception is the second survey where a large share of the participants completed the survey on the third day too. Potential reasons are: only on the third day, the Coronavirus press conference and the national TV news mentioned the survey and the invitation e-mail to the previous participants was only sent the evening of the second day.

Males participated less than females in all COVID-19 health surveys. There was an underrepresentation of the youngest (18-24 years) and oldest age groups (75+ years) in all COVID-19 health surveys. In addition throughout the time, a decline in the number of young participants (18-44 years) and an increase in the number of older participants (55-74 years) could be observed. There were also strong educational differences with, as expected, low educated people taking less part in the surveys. People from the Walloon Region were less prone to participate in the surveys. The recruitment approach of the COVID-19 health surveys did not make it possible to get (demographically) balanced samples. Other types of non-probability sampling approaches such as using paid and targeted adds on social media or retaining participants via commercial opt-in panels succeeded better in getting demographically balanced sample [13, 40].

### Limitations

The samples of the COVID-19 health surveys were prone to biased estimates as they relied on self-selection and excluded people without internet access or skills. This is the biggest point of criticism that non-probability web surveys receive [22, 23]. Despite of the efforts made, the unweighted sample distributions of the COVID-19 health surveys remained suboptimal. Post-stratification weighting on socio-demographic factors was applied to at least partly take into account the unequal distribution of some population groups in the COVID-19 health surveys. However, weighting for these factors is not sufficient to eliminate bias in the estimates. There are also unobservable characteristics which cannot be taken into account using weighting that impact both the chance to participate and the outcomes of the survey [23, 31].

As a consequence, caution is needed when generalizing results deriving from these type of non-probability web surveys to the general population. It is not recommended to calculate descriptive estimates such as prevalence rates from these surveys [29, 53, 54]. However, in the beginning of the pandemic there was an urgent need to have figures about the impact on the Belgian population. As there was no alternative in the form of a probability survey including people without internet access, the prevalence rates of the COVID-19 health surveys were considered as informative. Inferences regarding associations between variables are generally less sensitive to sampling quality [53]. Apart from the bias associated with the sampling, bias in the estimates can also result from the self-reporting aspect. For example, there might have been an overestimation of the compliance to preventive measures as this is a socially desirable behavior [55].

### Strenghts

The first asset is related to the questionnaire development and content. All surveys included as much as possible validated and frequently used instruments and scales. In addition, the surveys covered multiple health outcomes, highly relevant policy topics and contained a large set of covariates. The second major asset is the organization of a longitudinal study by re-inviting participants for next editions. A large share of participants completed the COVID-19 health surveys at least five times over two years (n cohort=12599). The benefit of following up the same individuals over time is that the evolution found for certain outcomes such as mental health throughout the pandemic cannot be due to different sample compositions across different time points [7]. The third major asset was the flexibility and timeliness to include new highly relevant topics in the surveys based on the demand of policy makers. The last asset is that the participants of the COVID-19 health surveys served as a recruitment pool for other COVID-19 projects including a qualitative study on the attitude towards vaccination.

### Future prospects and recommendations

The pandemic and the associated demand for data on the well-being of citizens taught us lessons for the future of survey methodology. In order to evaluate the impact of unexpected crises, we must ensure that we can survey randomly selected individuals instead of relying on convenience samples. Non-commercial online panels with a probability-based sample established prior to the crisis are an optimal choice for this [6, 21, 23, 36, 38]. Especially when providing panelists who do not have access to the internet with access to participate anyway or foreseeing them with paper response options. These studies limit self-selection bias and under-coverage bias and have valid comparison points with pre-crisis data. These types of panels did not exist in Belgium when the pandemic started but it is important to build them into our survey infrastructure. Fortunately, initiatives are currently taken to make up for this lack. There is, for instance, the “Belgian Health and Well-being cohort”, a cohort study initiated by Sciensano with a focus on mental health. This is the successor of the COVID-19 health surveys and the participant pool will consist of both previous participants of the COVID-19 health surveys and individuals selected from the national register. In addition to setting up large-scale panel studies, it is also relevant to always ask participants of large probability studies if they may be contacted by e-mail or postal mail in the future for follow-up research [6, 22].

The outcomes of the COVID-19 health surveys in terms of the participation and sample composition indicated that certain subgroups of the population are easy to attract for survey research and remain interested for follow-up surveys whereas for other subgroups the opposite holds. Also in probability surveys not organized in COVID-19 context, participation rates differ by socio-demographic characteristics [18, 56]. The large participation differences found in the COVID-19 health surveys made us think about using different recruitment approaches for different subgroups, especially for the youngsters. After consultations with internal communication experts, we started using different recruitment channels and different recruitment materials such as visuals for Instagram® to reach more youngster. Although the results were modest, experimenting with tailoring the data collection to different subgroups by using different recruitment materials, incentives or reminders instead of using a “one-method-fits-all-design” could be valuable. These type of studies have so-called adaptive or responsive survey designs [57, 58].

## Conclusion

These exceptional pandemic times have underlined the importance of collecting high quality data on people's experiences via surveys. However, traditional survey methodology was challenged in many ways in the beginning of the pandemic and, therefore, non-probability web surveys became an important information source. It is up to researchers involved in survey methodology to use these challenging times to improve the surveys organized in future crises times.

## Supplementary Information

**Additional file 1.** Checklist for reporting results of internet e-surveys.

## Data Availability

Access to the data reported in this manuscript is possible upon reasonable request by sending an e-mail to HIS@sciensano.be.

## References

[1] O’Connor DB, Aggleton JP, Chakrabarti B, Cooper CL, Creswell C, Dunsmuir S (2020). Research priorities for the COVID-19 pandemic and beyond: A call to action for psychological science. Br J Psychol.

[2] Holmes EA, O’Connor RC, Perry VH, Tracey I, Wessely S, Arseneault L (2020). Multidisciplinary research priorities for the COVID-19 pandemic: a call for action for mental health science. Lancet Psychiatry.

[3] Xiong J, Lipsitz O, Nasri F, Lui LMW, Gill H, Phan L (2020). Impact of COVID-19 pandemic on mental health in the general population: A systematic review. J Affect Disord.

[4] Armour C, McGlinchey E, Butter S, McAloney-Kocaman K, McPherson KE (2021). The COVID-19 Psychological Wellbeing Study: Understanding the Longitudinal Psychosocial Impact of the COVID-19 Pandemic in the UK; a Methodological Overview Paper. J Psychopathol Behav Assess.

[5] Ali SH, Foreman J, Capasso A, Jones AM, Tozan Y, DiClemente RJ (2020). Social media as a recruitment platform for a nationwide online survey of COVID-19 knowledge, beliefs, and practices in the United States: methodology and feasibility analysis. BMC Med Res Methodol.

[6] Hlatshwako TG, Shah SJ, Kosana P, Adebayo E, Hendriks J, Larsson EC (2021). Online health survey research during COVID-19. Lancet Digit Health..

[7] Bruggeman H, Smith P, Berete F, Demarest S, Hermans L, Braekman E (2022). Anxiety and Depression in Belgium during the First 15 Months of the COVID-19 Pandemic: A Longitudinal Study. Behav Sci.

[8] Ammar A, Brach M, Trabelsi K, Chtourou H, Boukhris O, Masmoudi L (2020). Effects of COVID-19 Home Confinement on Eating Behaviour and Physical Activity: Results of the ECLB-COVID19 International Online Survey. Nutrients.

[9] Drieskens S, Berger N, Vandevijvere S, Gisle L, Braekman E, Charafeddine R (2021). Short-term impact of the COVID-19 confinement measures on health behaviours and weight gain among adults in Belgium. Arch Public Health..

[10] Balanzá-Martínez V, Atienza-Carbonell B, Kapczinski F, De Boni RB (2020). Lifestyle behaviours during the COVID-19 – time to connect. Acta Psychiatr Scand..

[11] Vandevijvere S, De Ridder K, Drieskens S, Charafeddine R, Berete F, Demarest S (2021). Food insecurity and its association with changes in nutritional habits among adults during the COVID-19 confinement measures in Belgium. Public Health Nutr.

[12] van Loenhout JAF, Vanderplanken K, Van den Broucke S, Aujoulat I (2022). COVID-19 measures in Belgium: how perception and adherence of the general population differ between time periods. BMC Public Health.

[13] Grow A, Perrotta D, Del Fava E, Cimentada J, Rampazzo F, Gil-Clavel S (2020). Addressing Public Health Emergencies via Facebook Surveys: Advantages, Challenges, and Practical Considerations. J Med Internet Res.

[14] Geldsetzer P (2020). Use of Rapid Online Surveys to Assess People’s Perceptions During Infectious Disease Outbreaks: A Cross-sectional Survey on COVID-19. J Med Internet Res.

[15] De Coninck D, d’Haenens L, Matthijs K (2020). Perceived vulnerability to disease and attitudes towards public health measures: COVID-19 in Flanders. Belgium. Personal Individ Differ..

[16] Betsch C, Wieler LH, Habersaat K (2020). Monitoring behavioural insights related to COVID-19. Lancet Lond Engl..

[17] Roberts C. Mixing modes of data collection in surveys: A methodological review. In: NCRM Methods Review Paper. ESRC National Centre for Research Methods; 2007. Report No.: NCRM/008. Available from: http://eprints.ncrm.ac.uk/418/1/MethodsReviewPaperNCRM-008.pdf.

[18] Braekman E, Demarest S, Charafeddine R, Drieskens S, Berete F, Gisle L (2022). Unit response and costs in web versus face-to-face data collection: comparison of two cross-sectional health surveys. J Med Internet Res.

[19] Bowling A (2005). Mode of questionnaire administration can have serious effects on data quality. J Public Health.

[20] Tourangeau R, Rips LJ, Rasinski K (2000). The psychology of survey response.

[21] Kalaycioglu O (2020). Guidance for research on the Covid-19 disease in times of pandemic. J Health Soc Sci..

[22] De Man J, Campbell L, Tabana H, Wouters E (2021). The pandemic of online research in times of COVID-19. BMJ Open.

[23] Schaurer I, Weiß B (2020). Investigating selection bias of online surveys on coronavirus-related behavioral outcomes: An example utilizing the GESIS Panel Special Survey on the Coronavirus SARS-CoV-2 Outbreak in Germany. Surv Res Methods..

[24] Pierce M, McManus S, Jessop C, John A, Hotopf M, Ford T (2020). Says who? The significance of sampling in mental health surveys during COVID-19. Lancet Psychiatry.

[25] Bethlehem J (2010). Selection Bias in Web Surveys. Int Stat Rev.

[26] Statbel. ICT-gebruik in huishoudens. Cited 2020 Mar 17. Available from: https://statbel.fgov.be/nl/themas/huishoudens/ict-gebruik-huishoudens#figures.

[27] Schnell R, Noack M, Torregroza S (2017). Differences in General Health of Internet Users and Non-users and Implications for the Use of Web Surveys. Surv Res Methods..

[28] Callegaro M, Manfreda KL, Vehovar V (2015). Web Survey Methodology.

[29] Cornesse C, Blom AG, Dutwin D, Krosnick JA, De Leeuw ED, Legleye S (2020). A Review of Conceptual Approaches and Empirical Evidence on Probability and Nonprobability Sample Survey Research. J Surv Stat Methodol..

[30] Lehdonvirta V, Oksanen A, Räsänen P, Blank G (2021). Social Media, Web, and Panel Surveys: Using Non-Probability Samples in Social and Policy Research. Policy Internet.

[31] Lorant V, Smith P, Van den Broeck K, Nicaise P (2021). Psychological distress associated with the COVID-19 pandemic and suppression measures during the first wave in Belgium. BMC Psychiatry.

[32] Vanderbruggen N, Matthys F, Van Laere S, Zeeuws D, Santermans L, Van den Ameele S (2020). Self-Reported Alcohol, Tobacco, and Cannabis Use during COVID-19 Lockdown Measures: Results from a Web-Based Survey. Eur Addict Res.

[33] Cruyt E, De Vriendt P, De Letter M, Vlerick P, Calders P, De Pauw R (2021). Meaningful activities during COVID-19 lockdown and association with mental health in Belgian adults. BMC Public Health.

[34] Constandt B, Thibaut E, De Bosscher V, Scheerder J, Ricour M, Willem A (2020). Exercising in Times of Lockdown: An Analysis of the Impact of COVID-19 on Levels and Patterns of Exercise among Adults in Belgium. Int J Environ Res Public Health.

[35] Cellini N, Conte F, De Rosa O, Giganti F, Malloggi S, Reyt M (2021). Changes in sleep timing and subjective sleep quality during the COVID-19 lockdown in Italy and Belgium: age, gender and working status as modulating factors. Sleep Med.

[36] Kühne S, Kroh M, Liebig S, Zinn S (2020). The Need for Household Panel Surveys in Times of Crisis: The Case of SOEP-CoV. Surv Res Methods..

[37] Daly M, Sutin AR, Robinson E (2020). Longitudinal changes in mental health and the COVID-19 pandemic: evidence from the UK Household Longitudinal Study. Psychol Med.

[38] Riepenhausen A, Veer IM, Wackerhagen C, Reppmann ZC, Köber G, Ayuso-Mateos JL (2022). Coping with COVID: risk and resilience factors for mental health in a German representative panel study. Psychol Med.

[39] McBride O, Murphy J, Shevlin M, Gibson-Miller J, Hartman TK, Hyland P (2021). Monitoring the psychological, social, and economic impact of the COVID-19 pandemic in the population: Context, design and conduct of the longitudinal COVID-19 psychological research consortium (C19PRC) study. Int J Methods Psychiatr Res.

[40] Kittel B, Kritzinger S, Boomgaarden H, Prainsack B, Eberl JM, Kalleitner F (2021). The Austrian Corona Panel Project: monitoring individual and societal dynamics amidst the COVID-19 crisis. Eur Polit Sci.

[41] Eysenbach G. Improving the Quality of Web Surveys: The Checklist for Reporting Results of Internet E-Surveys (CHERRIES). J Med Internet Res. 2004;6(3):e34. 10.2196/jmir.6.3.e34. Erratum in: 10.2196/jmir.2042. 10.2196/jmir.6.3.e34 PMC1550605 15471760

[42] Sciensano: Belgium COVID-19 Dashboard. Cited 2022 Jun 15. Available from: https://datastudio.google.com/embed/reporting/c14a5cfc-cab7-4812-848c-0369173148ab/page/ZwmOB.

[43] Belgium.be: Overlegcomité keurt coronabarometer goed: code rood vanaf 28 januari. Cited 2022 Jul 14. Available from: https://www.belgium.be/nl/nieuws/2022/overlegcomite_keurt_coronabarometer_goed_code_rood_vanaf_28_januari.

[44] Belgium.be: Wat zijn de huidige maatregelen? | Coronavirus COVID-19. Cited 2022 Jun 17. Available from: https://www.info-coronavirus.be/nl/faq/.

[45] Sharma G (2017). Pros and cons of different sampling techniques. Int J Appl Res.

[46] European Commission, Eurostat. Labour Force Survey in the EU, candidate and EFTA countries: main characteristics of national surveys, 2018 : 2019 edition. Publications Office; 2019. Cited 2022 Jul 14. Available from: https://data.europa.eu/doi/10.2785/843203.

[47] Be.STAT: Bevolking naar woonplaats, nationaliteit (Belg/niet-Belg), burgerlijke staat, leeftijd en geslacht. Cited 2022 Jun 20. Available from: https://bestat.statbel.fgov.be/bestat/crosstable.xhtml?view=5fee32f5-29b0-40df-9fb9-af43d1ac9032.

[48] Be.STAT: Actieve (werkende en werkloze) en inactieve bevolking sinds 2017 op basis van de Enquête naar de ArbeidsKrachten, per jaar, gewest, leeftijdsklasse en onderwijsniveau. Cited 2022 Jun 20. Available from: https://bestat.statbel.fgov.be/bestat/crosstable.xhtml?view=631b4535-7a63-4695-967f-fe42238ee9af.

[49] Kim KS. Impact of Covid-19 on Survey Methods and Challenges. Am J Biomed Sci & Res. 2021;14(4). AJBSR. MS.ID.002011. 10.34297/AJBSR.2021.14.002011.

[50] Gummer T, Schmiedeberg C, Bujard M, Christmann P, Hank K, Kunz T, et al. The impact of Covid-19 on fieldwork efforts and planning in pairfam and FReDA-GGS. Surv Res Methods. 2020;14(2):223-227.

[51] Burton J, Lynn P, Benzeval M. How Understanding Society: The UK Household Longitudinal Study adapted to the COVID-19 pandemic. Surv Res Methods. 2020;14(2):235-239.

[52] Sastry N, McGonagle K, Fomby P (2020). Effects of the COVID-19 crisis on survey fieldwork: Experience and lessons from two major supplements to the U.S. Panel Study of Income Dynamics. Surv Res Methods..

[53] Pasek J (2016). When will Nonprobability Surveys Mirror Probability Surveys? Considering Types of Inference and Weighting Strategies as Criteria for Correspondence. Int J Public Opin Res..

[54] Kohler U, Kreuter F, Stuart EA (2019). Nonprobability Sampling and Causal Analysis. Annu Rev Stat Its Appl..

[55] Daoust JF, Nadeau R, Dassonneville R, Lachapelle E, Bélanger É, Savoie J (2021). How to Survey Citizens’ Compliance with COVID-19 Public Health Measures: Evidence from Three Survey Experiments. J Exp Polit Sci..

[56] Hermans L, Braekman E, Drieskens S, Demarest S (2022). Organizing the health interview survey at the local level: design of a pilot study. Arch Public Health..

[57] Tourangeau R, Michael Brick J, Lohr S, Li J (2017). Adaptive and responsive survey designs: A review and assessment. J R Stat Soc Ser A Stat Soc.

[58] Schouten B, Calinescu M, Luiten A (2013). Optimizing quality of response through adaptive survey designs. Surv Methodol.

