# Supplementary material for: Data collection in pandemic times: the case of the Belgian COVID-19 health surveys
Source: Arch Public Health. 2023 Jul 4;81:124. doi: 10.1186/s13690-023-01135-x (PMC10318627; doi:10.1186/s13690-023-01135-x)
Supplement: Supplementary file 1 — Additional file 1. Checklist for reporting results of internet e-surveys. [file 13690_2023_1135_MOESM1_ESM.docx]

# Checklist for Reporting Results of Internet E-Surveys (CHERRIES)

| ***Item Category*** | ***Checklist Item*** | ***Explanation*** |
| --- | --- | --- |
|  |  |  |
| Design | Describe survey design | The target population was the population of Belgium aged 18 years and older. The only exception was the first survey with the lower age limit of 16 years.  Non-probability convenience samples were used. |
| IRB (Institutional Review Board) approval and informed consent process | IRB approval | Approvals were received from the Ethics Committee of the Ghent University Hospital. |
|  | Informed consent | Through the welcome screen of the web questionnaires potential participants were informed about the length of the survey, the principal investigator, the purpose of the study, the confidentiality of the data, etc. Before being able to start the survey completion, respondents had to indicate that they agreed with the terms and conditions of the study. |
|  | Data protection | All data stored at Sciensano is saved on secure servers and access is limited to scientists who have an interest in the study. |
| Development and re-testing | Development and testing | The questionnaires were developed in LimeSurvey® version 3  Before each launch, the web questionnaire was tested by questionnaire experts. |
| Recruitment process and description of the sample having access to the questionnaire | Open survey versus closed survey | Open surveys: each adult with residence in Belgium could complete the survey. |
|  | Contact mode | The contacts were mainly via the internet, for some surveys there was also an offline recruitment via the Coronavirus press conferences or through a message on the television news or in paper journals |
|  | Advertising the survey | Online advertising   - Advertising on the website and social media of Sciensano of all surveys - Advertising on the website and social media of Belgian media organizations, local community organization, health insurance funds, elderly organizations, sports federations, higher education institutes and young adult organizations of most surveys (note: this was requested to these organizations, not obliged) - Advertising via an e-mail to previous participants starting from the second survey.   Offline advertising   - Advertising on Coronavirus press conferences of some surveys - Advertising on television news or in paper journals of some surveys |
| Survey administration | Web/E-mail | A web survey |
|  | Context | The surveys were published on a broad selection of websites (see the section “Advertising the survey”). This was done to avoid that our survey would only be completed by people with the same profile. |
|  | Mandatory/voluntary | The survey was voluntary. |
|  | Incentives | No incentives were offered. |
|  | Time/Date | The ten surveys were organized between April 2, 2020 and March 27, 2022. |
|  | Randomization of items or questionnaires | No randomization or alternation of questionnaire items or questionnaires was applied. |
|  | Adaptive questioning | To reduce the number of questions and complexity of the questionnaire adaptive questioning was applied. |
|  | Number of Items | Survey 1, 2 and 3 had the format ‘question by question’, meaning 1 question per page  From survey 4 on, we chose to use the format ‘Group by group’, meaning all the questions of a group per page. |
|  | Number of screens (pages) | Survey 1: 54 pages  Survey 2: 59 pages  Survey 3: 57 pages  Survey 4: 21 pages  Survey 5: 20 pages  Survey 6: 23 pages  Survey 7: 15 pages  Survey 8: 14 pages  Survey 9: 18 pages  Survey 10: 17 pages |
|  | Completeness check | A non-response option was foreseen for every question (always “I prefer not to say” and sometimes ‘Not applicable” or “I don’t know”) but respondents were not enforced to provide answers to all questions. No consistency or completeness checks were done before the questionnaire was submitted. |
|  | Review step | A “back” button was provided such that participants could change a previously given answer. A review step which displays a summary of the responses and asks the respondents if they are correct was not included. |
| Response rates | Unique site visitor | This was not foreseen. |
|  | View rate (Ratio of unique survey visitors/unique site visitors) | We were not able to calculate the view rates as the number of unique visitors could not be assessed. Moreover the surveys were published on multiple websites, including external organizations. |
|  | Participation rate (Ratio of unique visitors who agreed to participate/unique first survey page visitors) | We were not able to calculate the participation rate as we were unable to assess the number of unique visitors. |
|  | Completion rate (Ratio of users who finished the survey/users who agreed to participate) | Survey 1: 84,6%  Survey 2: 88,2%  Survey 3: 85,0%  Survey 4: 83,9%  Survey 5: 85,2%  Survey 6: 90,3%  Survey 7: 85,7%  Survey 8: 80,0%  Survey 9: 75,3%  Survey 10: 91,0% |
| Preventing multiple  entries from  the same  individual | Cookies used | No cookies were used. |
|  | IP check | IP addresses of the participant’s computers, smartphones or tablets were not used to identify potential duplicate entries from the same user. |
|  | Log file analysis | Log file analysis were not used to assess multiple entries. |
|  | Registration | Not applicable since it concerns an open survey. |
| Analysis | Handling of incomplete questionnaires | We only assessed the answers of participants replying to the questions on gender, age, municipality code and education level. |
|  | Questionnaires submitted with an atypical timestamp | We measured the time stamp at the beginning and ending of the questionnaire completion, but people with atypical timestamps were not by default excluded. |
|  | Statistical correction | Weighting methods were applied to adjust for the unequal sample distribution compared to the population distribution in terms of age group, sex, education level and region. |

Eysenbach G. Improving the Quality of Web Surveys: The Checklist for Reporting Results of Internet E-Surveys (CHERRIES). J Med Internet Res. 2004 Sep 29;6(3):e34. doi: 10.2196/jmir.6.3.e34. Erratum in: doi:10.2196/jmir.2042.
